# Safety and efficacy of laparoscopic sleeve gastrectomy post simultaneous kidney and pancreas transplant

**DOI:** 10.1093/jscr/rjae742

**Published:** 2024-11-24

**Authors:** Awadh Alqahtani, Mohammad Almayouf, Srikar Billa, Omar Alsarraj

**Affiliations:** Department of Surgery, College of Medicine, King Saud University, King Khalid road, P.O. Box 145111, Riyadh 4545, Saudi Arabia; Department of Surgery, College of Medicine, Prince Sattam bin Abdulaziz University, Abdullah bin Amer Road, P.O. Box 173, Alkharj 11942, Saudi Arabia; Department of Surgery, Sulaiman Al-Habib Hospitals, Takhassusi Road—Rahmaniya, P.O. Box 2000, Riyadh 11393, Saudi Arabia; Department of Surgery, Sulaiman Al-Habib Hospitals, Takhassusi Road—Rahmaniya, P.O. Box 2000, Riyadh 11393, Saudi Arabia

**Keywords:** obesity, bariatric surgery, kidney transplant, pancreas transplant, diabetes mellitus, renal failure

## Abstract

Few reports in the literature explore the efficacy of bariatric surgery in patients post simultaneous kidney and pancreas transplant (SKPT). This case report entails a patient who had SKPT because of end-stage renal disease and type I diabetes. The transplant failed, and the patient gained weight. The report explains the approach and the outcome of laparoscopic sleeve gastrectomy in this patient as a preparation for a re-transplant. The patient was having grade 2 obesity, insulin-dependent, on hemodialysis, and had obstructive sleep apnea on bilevel positive airway pressure. A multidisciplinary team approach was implemented, and the procedure was completed with no immediate postoperative complications. The patient lost ~10 kg and was able to stop insulin.

## Introduction

The negative effect of obesity on organ function is well documented in the literature. Glomerular hyperfiltration, hypertension, and insulin resistance syndrome are some of the mechanisms caused by obesity that can trigger or even accelerate chronic kidney disease progression [1]. Kidney damage can also be caused because of the overall inflammatory status caused by obesity [2]. Increased insulin demand provoked by increasing weight could increase the susceptibility of pancreatic B-cells to stress and autoimmune damage, thus increasing the chance of type-I diabetes development, especially in childhood [3]. Weight loss would reduce these damaging effects of obesity, and bariatric surgery is the most effective method to achieve sustainable weight loss and manage obesity-related diseases [4, 5].

Transplant candidates who have excessive weight are encouraged to lose weight and should be managed by all means that are appropriate as this would improve the transplant outcome. There is enough evidence in the literature supporting the positive effects of bariatric surgery as a weight loss strategy toward transplant, especially kidney transplant, but not enough data on multiorgan transplants [6]. This report aims to share the short-term outcome of bariatric surgery, specifically laparoscopic sleeve gastrectomy (LSG), in a post-multi organ transplant that failed and planned for a re-transplant.

## The case

Our patient is a 20-year-old male with morbid obesity (weight 94 kg, BMI 38.63 kg/m^2^), hypertension, end-stage renal disease (ESRD) due to juvenile nephrolithiasis/diabetic nephropathy on hemodialysis, long-standing type-I diabetes mellitus, obstructive sleep apnea on bilevel positive airway pressure (BiPAP), limited mobility because of bilateral slipped femoral capital epiphysis. His surgical history was significant for simultaneous kidney and pancreas transplant (SKPT) 4 years ago. The weight before the transplant was 68 kg, and the BMI of 27.58 kg/m^2^. The post-transplant period was significant for right subclavian venous thrombosis, pulmonary chylothorax, and sepsis requiring antibiotics. Insulin requirement improved, and there was no hemodialysis. The patient started to gain weight due to multiple factors, including his medication (5–10 mg prednisone daily, immunosuppressive medications), limited mobility, and poor dietary habits. Unfortunately, the transplanted kidney function deteriorated, because of BK virus-associated nephropathy. Additionally, his pancreatic function declined because of weight gain and developed type II diabetes because he had picture of hyperinsulinemia with elevated C-peptide. The patient resumed hemodialysis, increased insulin requirements, and reached grade III obesity. Non-operative obesity management using semaglutide was initiated but was not tolerable due to nausea and vomiting.

### Preoperative workup

After multiple meetings with the patient and his family and several consultations with different specialties, a consensus was reached that surgical management of the patient’s obesity is the best option as a staged procedure to prepare him for re-transplantation. A multidisciplinary team was involved in the patient’s preoperative workup.

**Bariatric surgeon:** Since the patient has grade III morbid obesity with multiple obesity-related diseases, and there are no gastroesophageal symptoms reported by the patient, an LSG was suggested. It is a safe surgery, effective, with low morbidity. Additionally, since the patient has multiple medications for his condition, absorption, and pharmacokinetics will not be affected drastically compared to other malabsorptive procedures.

**Clinical dietician/behavioral therapist:** A qualified dietician highlighted instructions about a healthy lifestyle, specifically diet. The dietician also addressed diet progression after LSG, education about high-quality foods, and eating habits (small portions, chewing, avoiding sugary beverages).

**Nephrology:** The recommendation for LSG was endorsed by the primary nephrologist as weight loss will benefit the patient significantly in preparation for a re-transplant. The immunosuppressive drug (Tacrolimus) and a small dose of prednisone were kept in the perioperative period to prevent the development of antibodies for future transplant. A stress dose of 50 mg hydrocortisone preoperatively, then continuing on a similar dose, was recommended. Hemodialysis sessions were planned before and after surgery.

**Cardiology:** Evaluation with echocardiography showed preserved left ventricular function. There was no evidence of valvular heart disease. A Dipyridamole stress test showed global hypokinesia with an ejection fraction of 44%. According to the modified Lee score, the patient was labeled as moderate risk. Postoperative intensive care unit monitoring was recommended.

**Hematology:** The patient had a history of right proximal subclavian vein thrombosis and was on direct oral anticoagulant (Apixaban 5 mg BID) previously. He was considered to have a high risk for thrombosis because of previous history of thrombosis, limited mobility, scheduled laparoscopic surgery, and ESRD. During the admission period, the choice of unfractionated heparin, and then switch to vitamin-K antagonist postoperatively was decided.

**Endocrinology:** The patient’s insulin regimen was 18 units of long-acting insulin and 4–8 units of pre-meal short-acting insulin. The preoperative glycated hemoglobin was 11.2%, so insulin doses were adjusted to achieve a lower level. In the postoperative period, an insulin sliding scale using long and short acting and insulin was planned with frequent glucose level monitoring during the admission period and after discharge.

**Pulmonology:** Considering the patient’s history of chylothorax and the requirement for BiPAP, a pulmonologist was requested to evaluate the patient. The chest X-ray showed no concerning pathology. The plan was to continue on BiPAP and postoperative chest physiotherapy.

### The operation

After following the recommendation from the involved subspecialties, the patient was brought to the operating room. A cardiac anesthesiologist induced the anesthesia. The procedure was conducted by a fellow of the Royal College of Physicians and surgeons of Canada trained surgeons. Patient positioning, pneumatic compression device application, and antibiotic prophylaxis administration were done. The abdomen was entered using 5 mm Vesiport™ superior and to the left of the umbilicus. Other ports were inserted with no issues. There were adhesions post-transplant, but not in the field of interest. A Nathanson liver retractor at the epigastric area was placed for better visualization of the stomach. The greater omentum was divided to the gastroesophageal junction, followed by applying staplers along a 36Fr bougie. After assuring hemostasis, plicating the staple line using monofilament, in addition to gastroepxy, is completed.

### Postoperative period

Following an established protocol, clear liquid intake was resumed 6 hours after surgery. The patient was kept at the intensive care unit for close monitoring for the first day. An established insulin sliding scale was started to achieve a glucose level target of 120–180 mg/dl. Heparin infusion was resumed 6 hours after surgery, in addition to the vitamin K antagonist, till reaching the targeted international normalized ratio. BiPAP was applied during sleep time. After discharge, the postoperative period was significant for hypoglycemic attacks, which required the endocrinologist to hold the insulin regimen as his random blood sugar readings were within normal limits. The follow-up appointment with the involved subspecialties was uneventful except for adjusting the vitamin K antagonist dose. Within 1 month, the patient lost 12 kg and was able to stop using the BiPAP.

## Discussion

Obesity can be a risk factor for poor outcomes in transplant patients, including rejection or subclinical rejection. Some of these factors contributing to complications in patients with obesity include obesity-related comorbidities in addition to inferior pharmacokinetics of immunosuppressive medications because of increased fat tissue mass [7, 8]. Weight gain following SKPT has been reported before, and the cause seems to be multifactorial. Although reports showed that there is no apparent risk for graft loss or loss of function, there is a higher chance of metabolic syndrome development [9]. Excessive weight and high insulin doses are independent factors for type II diabetes development following SKPT. Hence, weight loss strongly predicts positive outcomes following transplant, and bariatric surgery is the most effective tool available in obesity management [10]. This effectiveness was evident in our patient, who had a remarkable improvement in different aspects. The need for exogenous insulin was diminished in the early postoperative period, in addition to cessation of BiPAP usage. Weight loss was in the first month was 10 kg.

Scarce reports in the literature mentioned the bariatric surgery experience following failed SPKT. Zelones et al. reported a 52-year-old male patient undergoing LSG after SPKT with multiple comorbidities. The outcome was positive for lower insulin requirement, a better HbA1c level, and a weight loss of ~10 kg over 4 months [11]. Another report by Bonatti et al. reported the outcome of the application of an adjustable gastric band (AGB) in an SPKT patient, with minimum weight loss followed by weight regain after only 6 months [12]. We advise against the application of AGB as it is a foreign body, which can increase the chance of infectious complications in an immunocompromised patient.

## Conclusion

This report highlights the successful approach of a morbid transplant patient with obesity who underwent LSG. A multidisciplinary approach and a highly trained team are required in such cases.
